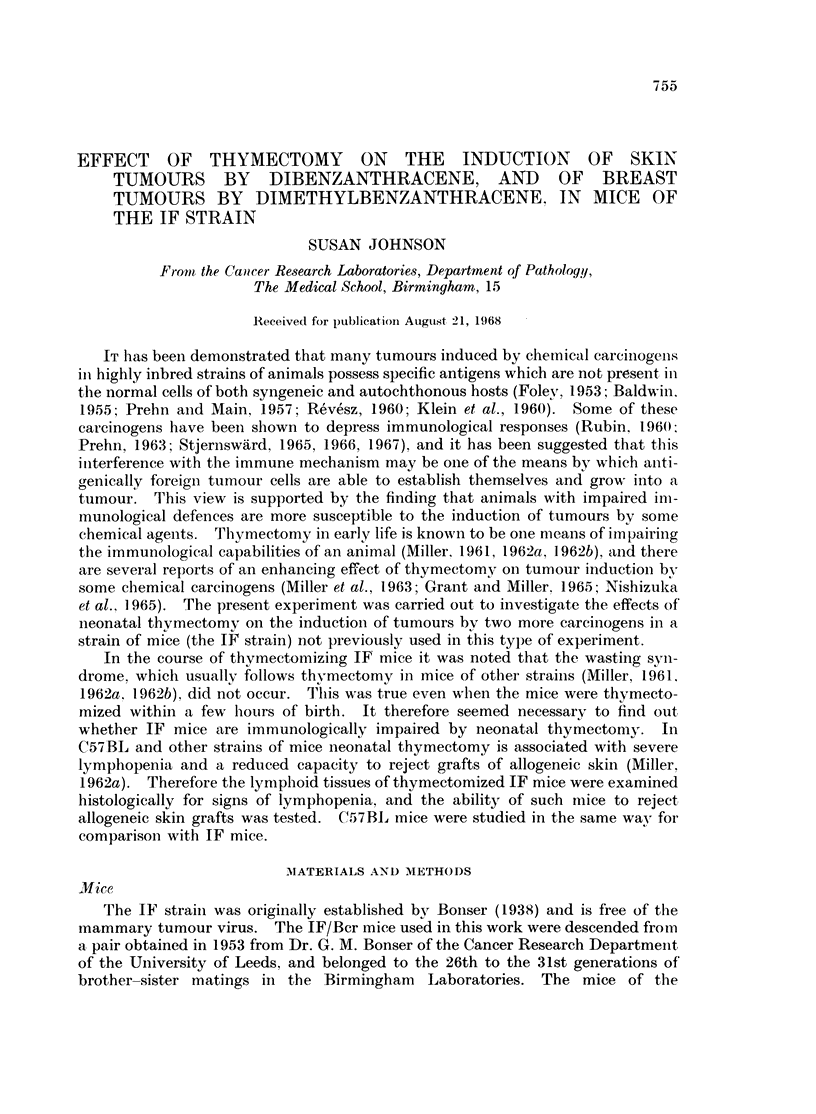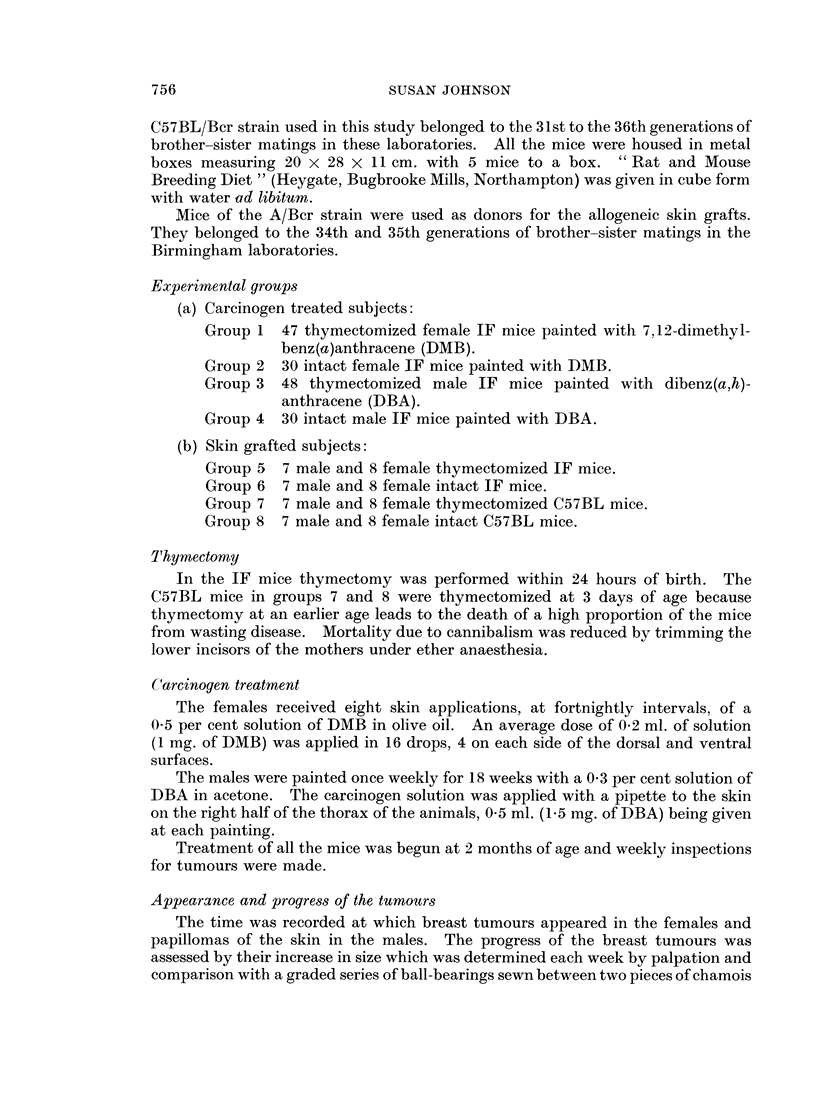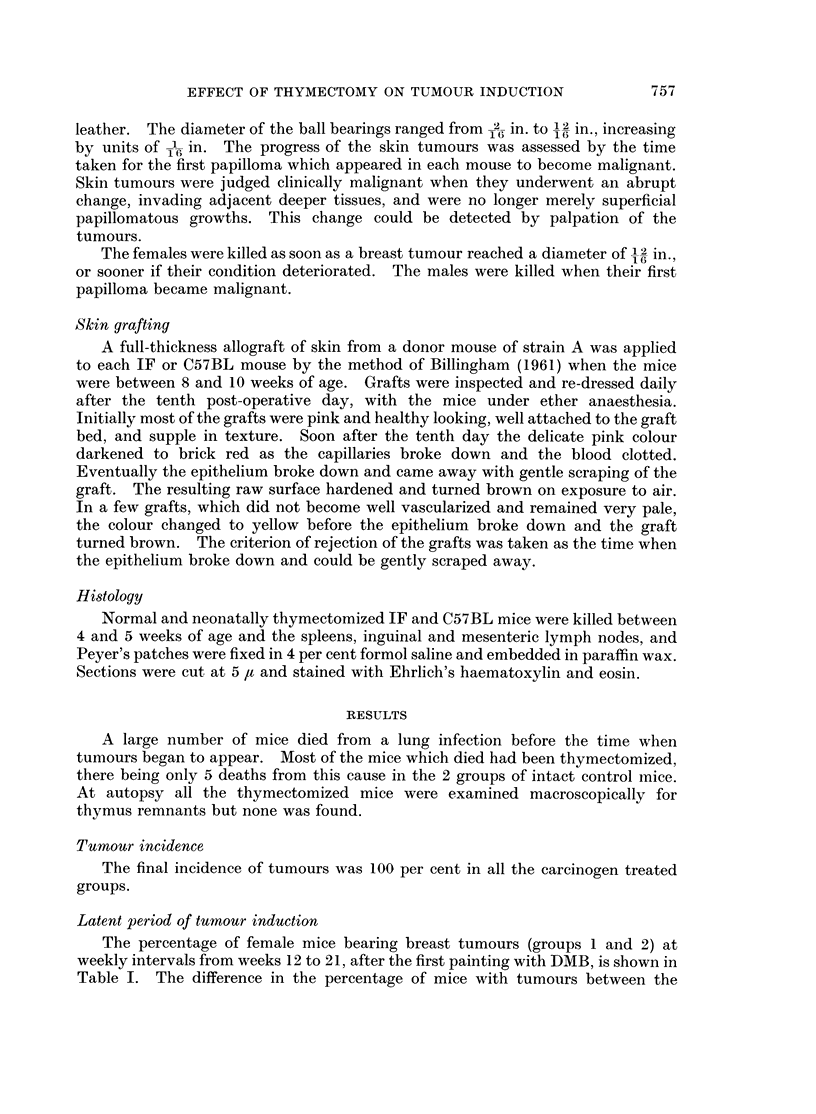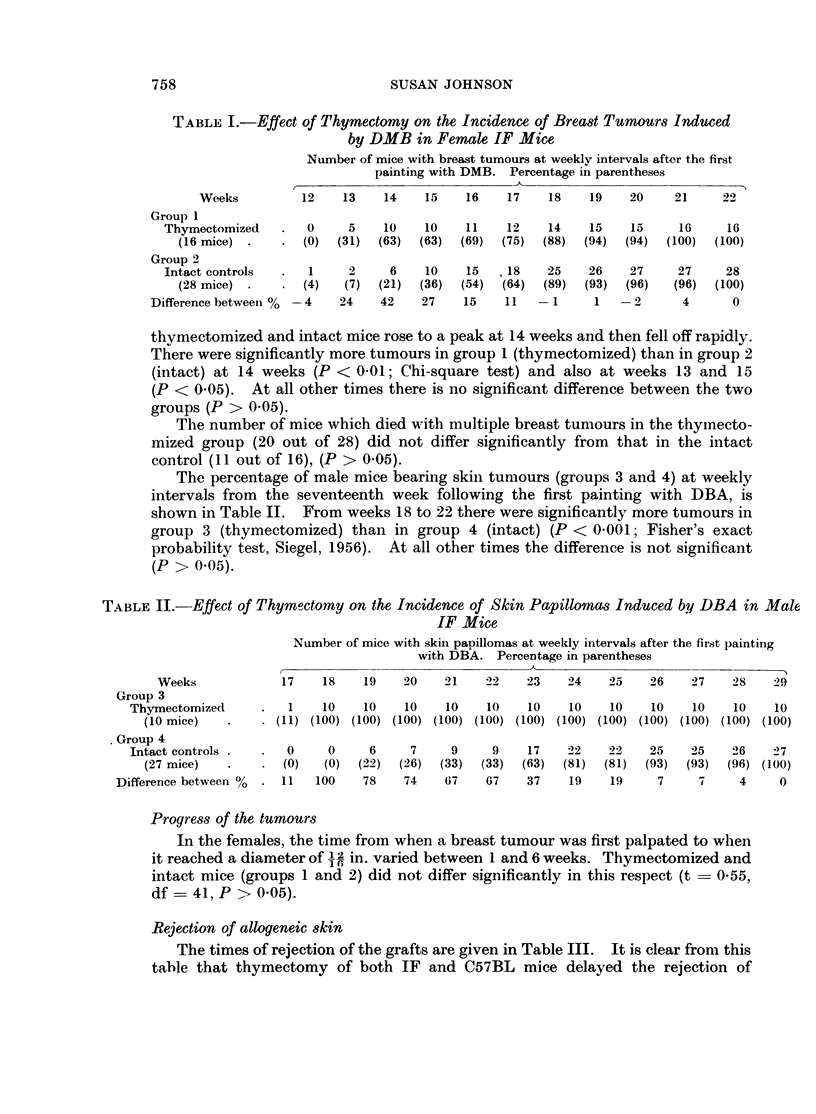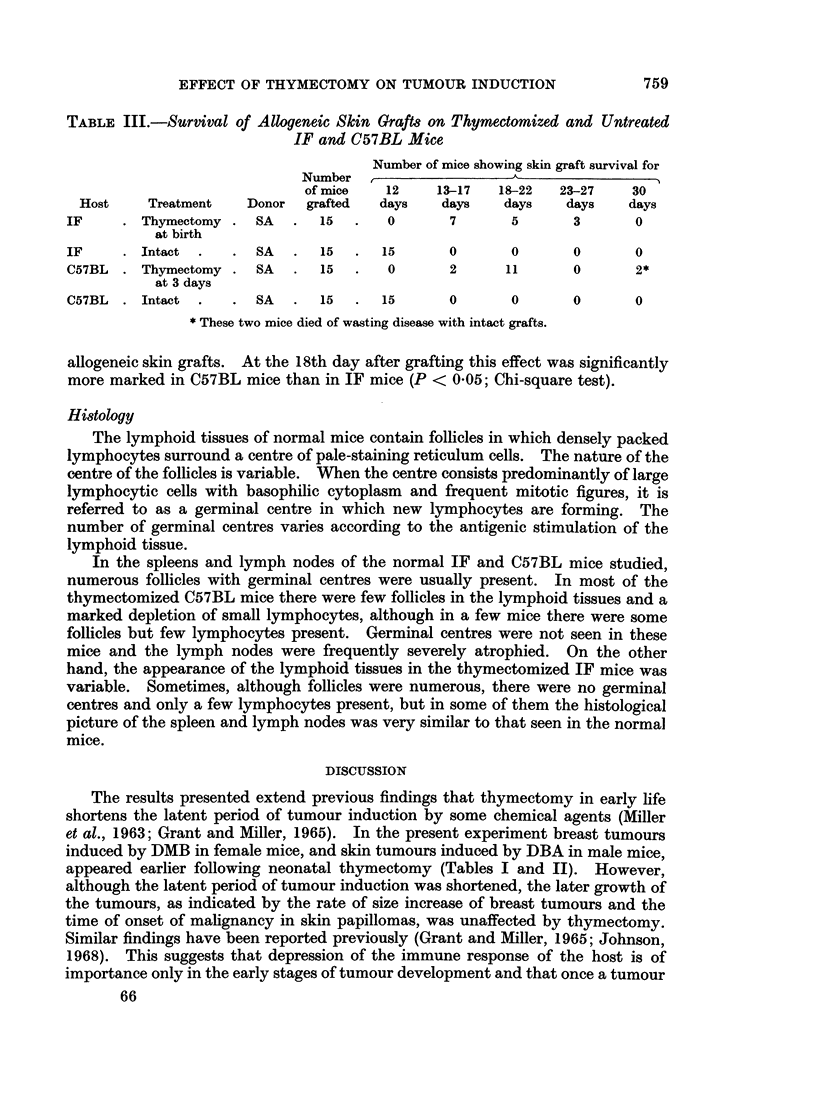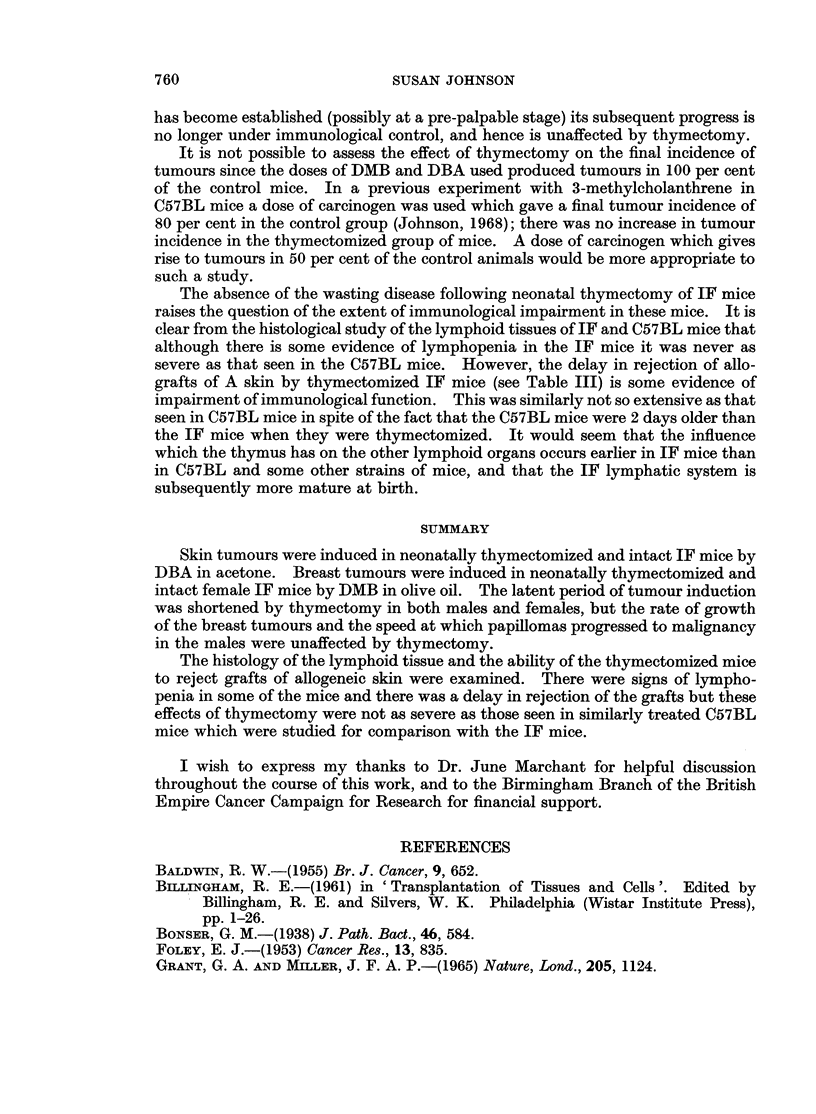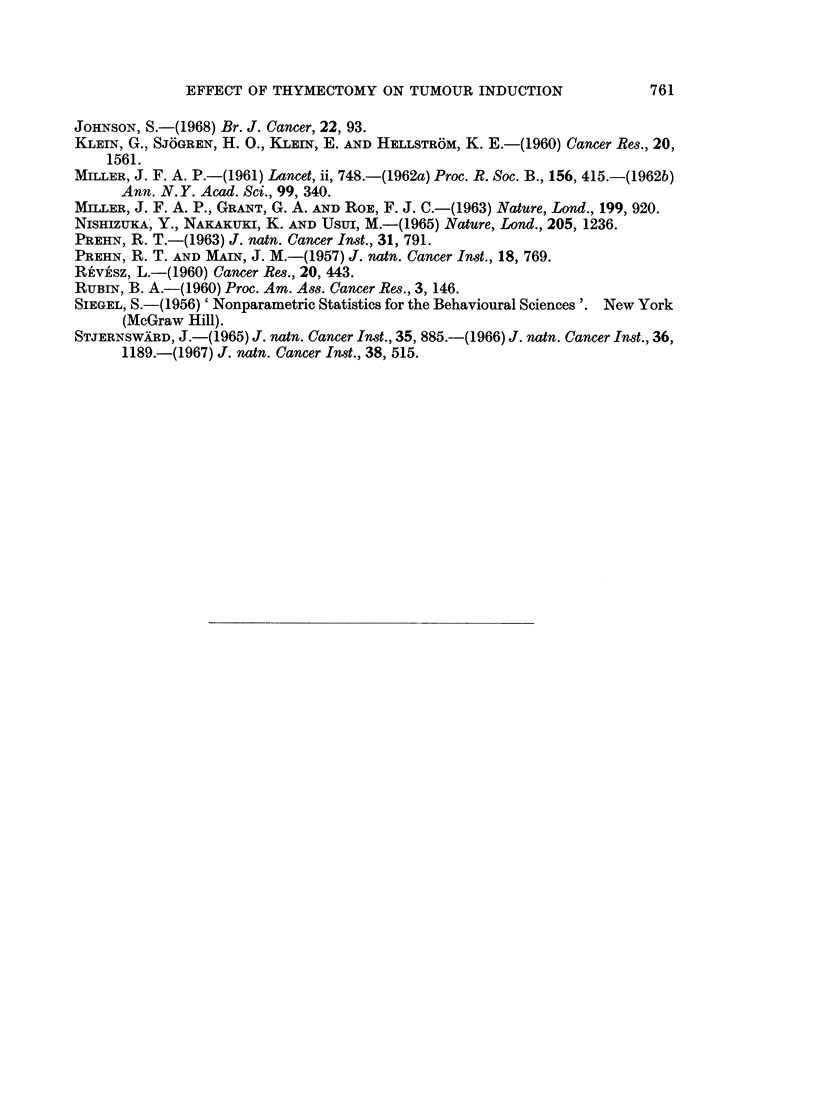# Effect of thymectomy on the induction of skin tumours by dibenzanthracene, and of breast tumours by dimethylbenzanthracene, in mice of the IF strain.

**DOI:** 10.1038/bjc.1968.89

**Published:** 1968-12

**Authors:** S. Johnson


### EFFECT OF THYMECTOMY ON THE INDUCTION OF SKIN TUMOURS BY DIBENZANTHRACENE, AND OF BREAST TUMOURS BY DIMETHYLBENZANTHRACENE, IN MICE OF THE IF STRAIN

SUSAN JOHNSON

*From the Cancer Research Laboratories, Department of Pathology, The Medical School, Birmingham,* 15

Received for publication August 21, 1968

It has been demonstrated that many tumours induced by chemical carcinogens in highly inbred strains of animals possess specific antigens which are not present in the normal cells of both syngeneic and autochthonous hosts (Foley, 1953; Baldwin, 1955; Prehn and Main, 1957; Révész, 1960; Klein *et al.*, 1960). Some of these carcinogens have been shown to depress immunological responses (Rubin, 1960; Prehn, 1963; Stjernswärd, 1965, 1966, 1967), and it has been suggested that this interference with the immune mechanism may be one of the means by which antigenically foreign tumour cells are able to establish themselves and grow into a tumour. This view is supported by the finding that animals with impaired immunological defences are more susceptible to the induction of tumours by some chemical agents. Thymectomy in early life is known to be one means of impairing the immunological capabilities of an animal (Miller, 1961, 1962*a*, 1962*b*), and there are several reports of an enhancing effect of thymectomy on tumour induction by some chemical carcinogens (Miller *et al.*, 1963; Grant and Miller, 1965; Nishizuka *et al.*, 1965). The present experiment was carried out to investigate the effects of neonatal thymectomy on the induction of tumours by two more carcinogens in a strain of mice (the IF strain) not previously used in this type of experiment.

In the course of thymectomizing IF mice it was noted that the wasting syndrome, which usually follows thymectomy in mice of other strains (Miller, 1961, 1962*a*, 1962*b*), did not occur. This was true even when the mice were thymectomized within a few hours of birth. It therefore seemed necessary to find out whether IF mice are immunologically impaired by neonatal thymectomy. In C57BL and other strains of mice neonatal thymectomy is associated with severe lymphopenia and a reduced capacity to reject grafts of allogeneic skin (Miller, 1962*a*). Therefore the lymphoid tissues of thymectomized IF mice were examined histologically for signs of lymphopenia, and the ability of such mice to reject allogeneic skin grafts was tested. C57BL mice were studied in the same way for comparison with IF mice.

### MATERIALS AND METHODS

### *Mice*

The IF strain was originally established by Bonser (1938) and is free of the mammary tumour virus. The IF/Bcr mice used in this work were descended from a pair obtained in 1953 from Dr. G. M. Bonser of the Cancer Research Department of the University of Leeds, and belonged to the 26th to the 31st generations of brother–sister matings in the Birmingham Laboratories. The mice of the

C57BL/Bcr strain used in this study belonged to the 31st to the 36th generations of brother–sister matings in these laboratories. All the mice were housed in metal boxes measuring 20 × 28 × 11 cm. with 5 mice to a box. "Rat and Mouse Breeding Diet" (Heygate, Bugbrooke Mills, Northampton) was given in cube form with water *ad libitum*.

Mice of the A/Bcr strain were used as donors for the allogeneic skin grafts. They belonged to the 34th and 35th generations of brother–sister matings in the Birmingham laboratories.

*Experimental groups*

(a) Carcinogen treated subjects:

Group 1 47 thymectomized female IF mice painted with 7,12-dimethylbenz(*a*)anthracene (DMB).
Group 2 30 intact female IF mice painted with DMB.
Group 3 48 thymectomized male IF mice painted with dibenz(*a*,*h*)-anthracene (DBA).
Group 4 30 intact male IF mice painted with DBA.

(b) Skin grafted subjects:

Group 5 7 male and 8 female thymectomized IF mice.
Group 6 7 male and 8 female intact IF mice.
Group 7 7 male and 8 female thymectomized C57BL mice.
Group 8 7 male and 8 female intact C57BL mice.

*Thymectomy*

In the IF mice thymectomy was performed within 24 hours of birth. The C57BL mice in groups 7 and 8 were thymectomized at 3 days of age because thymectomy at an earlier age leads to the death of a high proportion of the mice from wasting disease. Mortality due to cannibalism was reduced by trimming the lower incisors of the mothers under ether anaesthesia.

*Carcinogen treatment*

The females received eight skin applications, at fortnightly intervals, of a 0·5 per cent solution of DMB in olive oil. An average dose of 0·2 ml. of solution (1 mg. of DMB) was applied in 16 drops, 4 on each side of the dorsal and ventral surfaces.

The males were painted once weekly for 18 weeks with a 0·3 per cent solution of DBA in acetone. The carcinogen solution was applied with a pipette to the skin on the right half of the thorax of the animals, 0·5 ml. (1·5 mg. of DBA) being given at each painting.

Treatment of all the mice was begun at 2 months of age and weekly inspections for tumours were made.

*Appearance and progress of the tumours*

The time was recorded at which breast tumours appeared in the females and papillomas of the skin in the males. The progress of the breast tumours was assessed by their increase in size which was determined each week by palpation and comparison with a graded series of ball-bearings sewn between two pieces of chamois leather. The diameter of the ball bearings ranged from $\frac{2}{16}$ in. to $\frac{12}{16}$ in., increasing by units of $\frac{1}{16}$ in. The progress of the skin tumours was assessed by the time taken for the first papilloma which appeared in each mouse to become malignant. Skin tumours were judged clinically malignant when they underwent an abrupt change, invading adjacent deeper tissues, and were no longer merely superficial papillomatous growths. This change could be detected by palpation of the tumours.

The females were killed as soon as a breast tumour reached a diameter of $\frac{12}{16}$ in., or sooner if their condition deteriorated. The males were killed when their first papilloma became malignant.

*Skin grafting*

A full-thickness allograft of skin from a donor mouse of strain A was applied to each IF or C57BL mouse by the method of Billingham (1961) when the mice were between 8 and 10 weeks of age. Grafts were inspected and re-dressed daily after the tenth post-operative day, with the mice under ether anaesthesia. Initially most of the grafts were pink and healthy looking, well attached to the graft bed, and supple in texture. Soon after the tenth day the delicate pink colour darkened to brick red as the capillaries broke down and the blood clotted. Eventually the epithelium broke down and came away with gentle scraping of the graft. The resulting raw surface hardened and turned brown on exposure to air. In a few grafts, which did not become well vascularized and remained very pale, the colour changed to yellow before the epithelium broke down and the graft turned brown. The criterion of rejection of the grafts was taken as the time when the epithelium broke down and could be gently scraped away.

*Histology*

Normal and neonatally thymectomized IF and C57BL mice were killed between 4 and 5 weeks of age and the spleens, inguinal and mesenteric lymph nodes, and Peyer's patches were fixed in 4 per cent formol saline and embedded in paraffin wax. Sections were cut at 5 $\mu$ and stained with Ehrlich's haematoxylin and eosin.

### RESULTS

A large number of mice died from a lung infection before the time when tumours began to appear. Most of the mice which died had been thymectomized, there being only 5 deaths from this cause in the 2 groups of intact control mice. At autopsy all the thymectomized mice were examined macroscopically for thymus remnants but none was found.

*Tumour incidence*

The final incidence of tumours was 100 per cent in all the carcinogen treated groups.

*Latent period of tumour induction*

The percentage of female mice bearing breast tumours (groups 1 and 2) at weekly intervals from weeks 12 to 21, after the first painting with DMB, is shown in Table I. The difference in the percentage of mice with tumours between the

TABLE I.—*Effect of Thymectomy on the Incidence of Breast Tumours Induced by DMB in Female IF Mice*

| | Number of mice with breast tumours at weekly intervals after the first painting with DMB. Percentage in parentheses | | | | | | | | | | |
|---|---|---|---|---|---|---|---|---|---|---|---|
| Weeks | 12 | 13 | 14 | 15 | 16 | 17 | 18 | 19 | 20 | 21 | 22 |
| Group 1 | | | | | | | | | | | |
| Thymectomized | 0 | 5 | 10 | 10 | 11 | 12 | 14 | 15 | 15 | 16 | 16 |
| (16 mice) | (0) | (31) | (63) | (63) | (69) | (75) | (88) | (94) | (94) | (100) | (100) |
| Group 2 | | | | | | | | | | | |
| Intact controls | 1 | 2 | 6 | 10 | 15 | 18 | 25 | 26 | 27 | 27 | 28 |
| (28 mice) | (4) | (7) | (21) | (36) | (54) | (64) | (89) | (93) | (96) | (96) | (100) |
| Difference between % | −4 | 24 | 42 | 27 | 15 | 11 | −1 | 1 | −2 | 4 | 0 |

thymectomized and intact mice rose to a peak at 14 weeks and then fell off rapidly. There were significantly more tumours in group 1 (thymectomized) than in group 2 (intact) at 14 weeks ($P < 0{\cdot}01$; Chi-square test) and also at weeks 13 and 15 ($P < 0{\cdot}05$). At all other times there is no significant difference between the two groups ($P > 0{\cdot}05$).

The number of mice which died with multiple breast tumours in the thymectomized group (20 out of 28) did not differ significantly from that in the intact control (11 out of 16), ($P > 0{\cdot}05$).

The percentage of male mice bearing skin tumours (groups 3 and 4) at weekly intervals from the seventeenth week following the first painting with DBA, is shown in Table II. From weeks 18 to 22 there were significantly more tumours in group 3 (thymectomized) than in group 4 (intact) ($P < 0{\cdot}001$; Fisher's exact probability test, Siegel, 1956). At all other times the difference is not significant ($P > 0{\cdot}05$).

TABLE II.—*Effect of Thymectomy on the Incidence of Skin Papillomas Induced by DBA in Male IF Mice*

| | Number of mice with skin papillomas at weekly intervals after the first painting with DBA. Percentage in parentheses | | | | | | | | | | | | |
|---|---|---|---|---|---|---|---|---|---|---|---|---|---|
| Weeks | 17 | 18 | 19 | 20 | 21 | 22 | 23 | 24 | 25 | 26 | 27 | 28 | 29 |
| Group 3 | | | | | | | | | | | | | |
| Thymectomized | 1 | 10 | 10 | 10 | 10 | 10 | 10 | 10 | 10 | 10 | 10 | 10 | 10 |
| (10 mice) | (11) | (100) | (100) | (100) | (100) | (100) | (100) | (100) | (100) | (100) | (100) | (100) | (100) |
| Group 4 | | | | | | | | | | | | | |
| Intact controls | 0 | 0 | 6 | 7 | 9 | 9 | 17 | 22 | 22 | 25 | 25 | 26 | 27 |
| (27 mice) | (0) | (0) | (22) | (26) | (33) | (33) | (63) | (81) | (81) | (93) | (93) | (96) | (100) |
| Difference between % | 11 | 100 | 78 | 74 | 67 | 67 | 37 | 19 | 19 | 7 | 7 | 4 | 0 |

*Progress of the tumours*

In the females, the time from when a breast tumour was first palpated to when it reached a diameter of $\frac{12}{16}$ in. varied between 1 and 6 weeks. Thymectomized and intact mice (groups 1 and 2) did not differ significantly in this respect ($t = 0{\cdot}55$, $df = 41$, $P > 0{\cdot}05$).

*Rejection of allogeneic skin*

The times of rejection of the grafts are given in Table III. It is clear from this table that thymectomy of both IF and C57BL mice delayed the rejection of

TABLE III.—*Survival of Allogeneic Skin Grafts on Thymectomized and Untreated IF and C57BL Mice*

| Host | Treatment | Donor | Number of mice grafted | Number of mice showing skin graft survival for 12 days | 13–17 days | 18–22 days | 23–27 days | 30 days |
|---|---|---|---|---|---|---|---|---|
| IF | Thymectomy at birth | SA | 15 | 0 | 7 | 5 | 3 | 0 |
| IF | Intact | SA | 15 | 15 | 0 | 0 | 0 | 0 |
| C57BL | Thymectomy at 3 days | SA | 15 | 0 | 2 | 11 | 0 | 2* |
| C57BL | Intact | SA | 15 | 15 | 0 | 0 | 0 | 0 |

* These two mice died of wasting disease with intact grafts.

allogeneic skin grafts. At the 18th day after grafting this effect was significantly more marked in C57BL mice than in IF mice ($P < 0{\cdot}05$; Chi-square test).

*Histology*

The lymphoid tissues of normal mice contain follicles in which densely packed lymphocytes surround a centre of pale-staining reticulum cells. The nature of the centre of the follicles is variable. When the centre consists predominantly of large lymphocytic cells with basophilic cytoplasm and frequent mitotic figures, it is referred to as a germinal centre in which new lymphocytes are forming. The number of germinal centres varies according to the antigenic stimulation of the lymphoid tissue.

In the spleens and lymph nodes of the normal IF and C57BL mice studied, numerous follicles with germinal centres were usually present. In most of the thymectomized C57BL mice there were few follicles in the lymphoid tissues and a marked depletion of small lymphocytes, although in a few mice there were some follicles but few lymphocytes present. Germinal centres were not seen in these mice and the lymph nodes were frequently severely atrophied. On the other hand, the appearance of the lymphoid tissues in the thymectomized IF mice was variable. Sometimes, although follicles were numerous, there were no germinal centres and only a few lymphocytes present, but in some of them the histological picture of the spleen and lymph nodes was very similar to that seen in the normal mice.

### DISCUSSION

The results presented extend previous findings that thymectomy in early life shortens the latent period of tumour induction by some chemical agents (Miller *et al.*, 1963; Grant and Miller, 1965). In the present experiment breast tumours induced by DMB in female mice, and skin tumours induced by DBA in male mice, appeared earlier following neonatal thymectomy (Tables I and II). However, although the latent period of tumour induction was shortened, the later growth of the tumours, as indicated by the rate of size increase of breast tumours and the time of onset of malignancy in skin papillomas, was unaffected by thymectomy. Similar findings have been reported previously (Grant and Miller, 1965; Johnson, 1968). This suggests that depression of the immune response of the host is of importance only in the early stages of tumour development and that once a tumour has become established (possibly at a pre-palpable stage) its subsequent progress is no longer under immunological control, and hence is unaffected by thymectomy.

It is not possible to assess the effect of thymectomy on the final incidence of tumours since the doses of DMB and DBA used produced tumours in 100 per cent of the control mice. In a previous experiment with 3-methylcholanthrene in C57BL mice a dose of carcinogen was used which gave a final tumour incidence of 80 per cent in the control group (Johnson, 1968); there was no increase in tumour incidence in the thymectomized group of mice. A dose of carcinogen which gives rise to tumours in 50 per cent of the control animals would be more appropriate to such a study.

The absence of the wasting disease following neonatal thymectomy of IF mice raises the question of the extent of immunological impairment in these mice. It is clear from the histological study of the lymphoid tissues of IF and C57BL mice that although there is some evidence of lymphopenia in the IF mice it was never as severe as that seen in the C57BL mice. However, the delay in rejection of allografts of A skin by thymectomized IF mice (see Table III) is some evidence of impairment of immunological function. This was similarly not so extensive as that seen in C57BL mice in spite of the fact that the C57BL mice were 2 days older than the IF mice when they were thymectomized. It would seem that the influence which the thymus has on the other lymphoid organs occurs earlier in IF mice than in C57BL and some other strains of mice, and that the IF lymphatic system is subsequently more mature at birth.

### SUMMARY

Skin tumours were induced in neonatally thymectomized and intact IF mice by DBA in acetone. Breast tumours were induced in neonatally thymectomized and intact female IF mice by DMB in olive oil. The latent period of tumour induction was shortened by thymectomy in both males and females, but the rate of growth of the breast tumours and the speed at which papillomas progressed to malignancy in the males were unaffected by thymectomy.

The histology of the lymphoid tissue and the ability of the thymectomized mice to reject grafts of allogeneic skin were examined. There were signs of lymphopenia in some of the mice and there was a delay in rejection of the grafts but these effects of thymectomy were not as severe as those seen in similarly treated C57BL mice which were studied for comparison with the IF mice.

I wish to express my thanks to Dr. June Marchant for helpful discussion throughout the course of this work, and to the Birmingham Branch of the British Empire Cancer Campaign for Research for financial support.

### REFERENCES

BALDWIN, R. W.—(1955) *Br. J. Cancer*, **9**, 652.

BILLINGHAM, R. E.—(1961) in 'Transplantation of Tissues and Cells'. Edited by Billingham, R. E. and Silvers, W. K. Philadelphia (Wistar Institute Press), pp. 1–26.

BONSER, G. M.—(1938) *J. Path. Bact.*, **46**, 584.

FOLEY, E. J.—(1953) *Cancer Res.*, **13**, 835.

GRANT, G. A. AND MILLER, J. F. A. P.—(1965) *Nature, Lond.*, **205**, 1124.

JOHNSON, S.—(1968) *Br. J. Cancer*, **22**, 93.
KLEIN, G., SJÖGREN, H. O., KLEIN, E. AND HELLSTRÖM, K. E.—(1960) *Cancer Res.*, **20**, 1561.
MILLER, J. F. A. P.—(1961) *Lancet*, ii, 748.—(1962*a*) *Proc. R. Soc.* B., **156**, 415.—(1962*b*) *Ann. N.Y. Acad. Sci.*, **99**, 340.
MILLER, J. F. A. P., GRANT, G. A. AND ROE, F. J. C.—(1963) *Nature, Lond.*, **199**, 920.
NISHIZUKA, Y., NAKAKUKI, K. AND USUI, M.—(1965) *Nature, Lond.*, **205**, 1236.
PREHN, R. T.—(1963) *J. natn. Cancer Inst.*, **31**, 791.
PREHN, R. T. AND MAIN, J. M.—(1957) *J. natn. Cancer Inst.*, **18**, 769.
RÉVÉSZ, L.—(1960) *Cancer Res.*, **20**, 443.
RUBIN, B. A.—(1960) *Proc. Am. Ass. Cancer Res.*, **3**, 146.
SIEGEL, S.—(1956) ' Nonparametric Statistics for the Behavioural Sciences '. New York (McGraw Hill).
STJERNSWÄRD, J.—(1965) *J. natn. Cancer Inst.*, **35**, 885.—(1966) *J. natn. Cancer Inst.*, **36**, 1189.—(1967) *J. natn. Cancer Inst.*, **38**, 515.